# IL-1β/HMGB1 Complexes Promote The PGE_2_ Biosynthesis Pathway in Synovial Fibroblasts

**DOI:** 10.1111/sji.12041

**Published:** 2013-04-23

**Authors:** P Leclerc, H Wähämaa, H Idborg, P J Jakobsson, H E Harris, M Korotkova

**Affiliations:** * Rheumatology Research Unit, Department of Medicine, Karolinska InstitutetStockholm, Sweden; † Pediatric Rheumatology Unit, Department of Woman and Child Health, Karolinska InstitutetStockholm, Sweden; ‡ Actar ABSolna, Sweden

## Abstract

PGE_2_ is a potent lipid mediator of pain and oedema found elevated in RA. Microsomal prostaglandin E synthase-1 (mPGES-1) is a terminal enzyme of the PGE_2_ pathway inducible by proinflammatory cytokines. mPGES-1 is markedly upregulated in RA synovial tissue despite antirheumatic treatments, suggesting that multiple inflammatory stimuli contribute to its induction. High-mobility group box chromosomal protein 1 (HMGB1) is known to induce inflammation both by direct interaction with TLR4 and by enhancement of other proinflammatory molecules signalling, through complex formation. The high expression of extracellular HMGB1 within the inflamed synovium, implies its pro-arthritogenic role in RA. We aimed to investigate the effects of IL-1β/HMGB1 complexes on mPGES-1 and other enzymes of the PGE_2_ pathway in synovial fibroblasts (SFs) from patients with arthritis. Furthermore, we studied the effect of COX-2 inhibition and IL-1RI antagonism on prostanoid and cytokine production by SFs. Stimulation of SFs with HMGB1 in complex with suboptimal amounts of IL-1β significantly increased mPGES-1 and COX-2 expressions as well as PGE_2_ production, as compared to treatment with HMGB1 or IL-1β alone. Furthermore, NS-398 reduced the production of IL-6 and IL-8, thus indicating that IL-1β/HMGB1 complexes modulate cytokine production in part through prostanoid synthesis. Treatment with IL-1RA completely abolished the induced PGE_2_ and cytokine production, suggesting an effect mediated through IL-1RI. IL-1β/HMGB1 complexes promote the induction of mPGES-1, COX-2 and PGE_2_ in SF. The amplification of the PGE_2_ biosynthesis pathway by HMGB1 might constitute an important pathogenic mechanism perpetuating inflammatory and destructive activities in rheumatoid arthritis.

## Introduction

Rheumatoid arthritis (RA) is a chronic inflammatory disease characterized by synovial inflammation as well as bone and cartilage destruction, leading to loss of joint function. In RA, the inflamed synovium produces elevated levels of PGE_2_, a powerful proinflammatory lipid mediator that triggers pain and oedema. PGE_2_ production/biosynthesis in inflammation is mainly regulated by the concerted activities of three enzymes: microsomal prostaglandin E synthase-1 (mPGES-1), cyclooxygenase-2 (COX-2) and 15-hydroxyprostaglandin dehydrogenase (15-PGDH). The former two enzymes are involved in the synthesis of PGE_2_ and are inducible by a range of proinflammatory stimuli such as IL-1β, TNF-α and IL-6 1, 2, whereas 15-PGDH is an enzyme degrading PGE_2_. The role of the inducible PGE_2_ pathway in RA has proven to be central as COX-2 selective inhibitors are an effective treatment for RA-associated pain and inflammation 3. mPGES-1 acts downstream from the cyclooxygenase enzymes and is responsible for the conversion of PGH_2_ into PGE_2_ 4. mPGES-1 is strongly upregulated in the RA synovium, making it a potential therapeutic target 5. Moreover, the expression of mPGES-1 in RA synovial tissue is not properly targeted by current antirheumatic treatments. In fact, TNF-α targeted therapy, B cell depletion therapy and methotrexate treatment all fail to inhibit the expression of mPGES-1 in the RA synovium, suggesting that multiple mechanisms are involved in the induction of mPGES-1 expression in the RA synovium 6–8.

High-mobility group box chromosomal protein 1 (HMGB1) is an alarmin possessing distinct functions intra- and extracellularly. In the cell nucleus, HMGB1 acts as a DNA-binding protein, participating in transcription, recombination and DNA repair. When released to the extracellular space, HMGB1 acts as an important mediator of inflammation, promoting both acute inflammation and subsequent tissue repair 9. We and other research groups have demonstrated that the inflammation-promoting effects of HMGB1 are in part mediated through its binding to TLR2 and TLR4 and require the HMGB1 molecule to be in a specific redox status 10, 11. However, regardless of its oxidation status, HMGB1 is also able to potently promote and enhance inflammation by complex formation with other proinflammatory molecules such as IL-1α/β, LPS, CpG-DNA, the TLR1/TLR2-agonist Pam3CSK4, nucleosomes and CXCL12 12–17. In rodent models of experimentally induced arthritis, HMGB1 is an essential mediator of inflammation and joint destruction as HMGB1-blocking therapies ameliorate both the inflammatory and tissue destructive disease course in collagen-induced arthritis and the spontaneous arthritis developing in DNAseII x IFNR1 deficient mice 18–22. In RA, extracellular HMGB1 is found within the inflamed synovial tissue and in the synovial fluid, implying its proarthritogenic role in RA 23. Moreover, we have recently demonstrated that HMGB1 in complex with suboptimal concentrations of IL-1α, IL-1β or LPS was able to induce inflammatory cytokine production from RA synovial fibroblasts (RASF) 24. Interestingly, like mPGES-1, HMGB1 levels in the RA synovium are reduced by intra-articular treatment with glucocorticoids, but remain unaltered in patients on anti-TNF therapy 25, 26, suggesting that HMGB1 might be a potential inducer of mPGES-1 in inflamed tissue.

The involvement of IL-1 in RA has previously been shown by clinical improvement in patients on IL-1 receptor antagonist (IL-1Ra) therapy, reducing both clinical signs of inflammation and joint erosion 27. IL-1β can stimulate synovial fibroblasts to release mediators promoting inflammation and cartilage/bone degradation 1, 24. In this study, we aimed to investigate the effects of IL-1β/HMGB1 complexes on mPGES-1 and other enzymes of the PGE_2_ pathway in SFs from patients with inflammatory arthritis. Furthermore, we studied the effect of COX-2 inhibition and IL-1RI antagonism on prostanoid and cytokine production by SFs.

## Materials and methods

### Preparation of rHMGB1 from *E. Coli*

Recombinant rat HMGB1 (rHMGB1), with a 99% identity to human HMGB1 28 and containing a calmodulin-binding protein tag, was expressed in *E. coli* strain BL21 (for sequence see 29). Protein was purified by sequential ion exchange chromatography (MonoS 5/50 GL column, GE Healthcare, Chalfont St. Giles, UK) and calmodulin affinity chromatography (Calmodulin sepharose 4B, GE Healthcare, Uppsala, Sweden). Endotoxin was removed by filtration through Acodisc Units with Mustang E Membranes (0.25 μm, Pall Life Sciences, East Hills, NY, USA), yielding endotoxin levels below 0.03 EU/μg protein, as measured by the Limulus assay. Preparations of HMGB1 in 20 mm 3-(N-Morpholino) propanesulphonic acid (MOPS), 400-mm NaCl, 20-mm EGTA, 10-mm dithiothreitol at pH 8.0 were stored at −80°C until day of use. The HMGB1 used in the studies did not induce cytokine production *per se*.

### Preparation of IL-1β/HMGB1 solutions

r/tHMGB1 and IL-1β were mixed in PBS. A 50X IL-1β/HMGB1 solution was prepared in a ratio allowing the indicated final concentrations in cell cultures after dilution. Solutions were incubated at 4°C for 16 h before addition to cell cultures. IL-1β/HMGB1 complex formation has previously been demonstrated 30.

### Cell cultures

Synovial fibroblasts were obtained from ten patients: two from RA patients were purchased from Asterand (Detroit, MI, USA), and eight were propagated from synovial tissues isolated from four RA and four juvenile idiopathic arthritis patients undergoing joint replacement surgery as previously described 31. This study was approved by the Institutional Ethical Committee (Solna, Stockholm, Sweden ethical number 2009/1262-31/3) and is in compliance with all ethical standards and patients′ consent according to the Declaration of Helsinki. Briefly, synovial tissues were minced, and explants were maintained in DMEM supplemented with 10% heat-inactivated FCS (PAA Laboratories, Linz, Austria), 100 U/ml penicillin, 100 μg/ml streptomycin and HEPES (Life Technologies, Paisely, Scotland, UK) (complete DMEM) in a tissue culture incubator at 37°C with 5% CO_2_ content. Tissue explants and non-adherent cells were discarded after 1–2 weeks of culture. Adherent cells were trypsinized with trypsin-EDTA (Gibco, Scotland, UK) at 80% confluence and used for experiments at passages 3–8. Synovial fibroblasts grown to confluence were trypsinized with trypsin-EDTA and washed with complete DMEM. Cell viability was assessed using trypan blue (Merck, Darmstadt, Germany) in every experimental set up and was 95–100%.

Cells were plated in 96-well plates at 4000 cells/well, 6-well plates at 160 000 cells/well or 10 cm Nunclon dishes at 800 000 cells/well and allowed to rest for 15–17 h in a tissue culture incubator at 37°C with 5% CO_2_ content. Medium was discarded, and cells were washed twice with OPTIMEM (Gibco, Scotland, UK) supplemented with 100 U/ml penicillin, 100 μg/ml streptomycin. Cell stimulations were carried in OPTIMEM. The cells were stimulated for 4–72 h with 100 ng/ml rHMGB1 or calf thymus-extracted (t)HMGB1 (kind gift from Dr. Michael Bustin, NIH) alone or in complex with 0.05 (10 donors) or sometimes with 0.5 (4 donors) ng/ml rIL-1β (R&D systems, Minneapolis, MN, USA) as indicated. The latter two concentrations of rIL-1β were referred to as IL-1β_low_ and were used in parallel because the threshold of response of SFs to IL-1 β alone was found to vary among experiments. The highest concentration of IL-1 β_low_ not eliciting a response from SF was used as a negative control to IL-1β_low_/HMGB1 complexes. 5 ng/ml rIL-1β (IL-1β_high_) was used as a positive control. In certain experiments, cells were pretreated for 1–2 h with 5 μg/ml IL-1 receptor antagonist (IL-1Ra/anakinra). NS-398 (Sigma-Aldrich, St.Louis, MO, USA), the COX-2 inhibitor, or vehicle alone (0.5% DMSO), was added at the concentration of 0.1 μM, simultaneously with inflammatory stimuli. Supernatants were collected at the indicated time points and stored at −70°C until analysis.

### Prostanoid analysis

PGE_2_ levels were measured by enzyme immunoassay (Cayman Chemicals, Ann Harbor, MI, USA) and normalized for cell viability using a MTT-based *in vitro* toxicology assay kit (Sigma- Aldrich). Prostaglandin profiling was performed by liquid chromatography coupled to tandem mass spectrometry (LC-MS/MS) on a Waters 2795 HPLC (Waters Corporation, Milford, MA, USA) coupled to a triple quadrupole mass spectrometer (Acquity TQ Detector, Waters Corporation). Cell culture supernatants were analysed for PGE_2_, PGD_2_, PGF_2α_, TXB_2_ and 6-keto-PGF_1α_. After addition of deuterated isomers of all analytes to 50 μl of sample, prostanoids were extracted on an Oasis HLB Extraction Plate (Waters Corporation). Extracted material was evaporated and reconstituted in 50 μl sample solvent (H_2_O, 7% acetonitrile (ACN), 0.05% formic acid (FA)), and 40 μl were injected. Separation of the analytes was achieved on a Synergi Hydro-RP column (100 mm x 2 mm i.d., 2.5 μm particle size and 100 Å pore size, Phenomenex, CA, USA) during a 45 min stepwise linear gradient using Milli-Q H_2_O as mobile phase A and ACN, 0.05% FA as mobile phase B. Concentration of mobile phase B was increased from 10% to 25% during 9 min in step 1, then to 45% during 22 min in step 2 and furthermore to 70% during 5 min in step 3, followed by a wash step at 90% mobile phase B and re-equilibration at 10% mobile phase B. The analytes were detected in multiple reaction monitoring (MRM) mode, recording the transitions of *m/z* 351.2 → *m/z* 271.2 for PGE_2_ and PGD_2_, *m/z* 353.2 → *m/z* 309.1 for PGF_2α_, *m/z* 369.2 → *m/z* 169.1 for TXB_2_ and *m/z* 369.3 → *m/z* 163.2 for 6-keto PGF_1α_. Analysis of the MRM data was carried out with MassLynx software, version 4.1, using an internal standard calibration curve of all analytes for quantification. When prostanoid concentrations were below the limit of quantification (LOQ), they were given an arbitrary value equal to the lowest quantifiable standard for statistical purpose.

### Western blot

Cells lysis was performed on ice (30 min) using tissue protein extraction reagent (T-PER) (Thermo Scientific, Rockford, IL, USA) supplemented with 1X complete protease inhibitor cocktail (Roche Diagnostics GmbH, Mannheim, Germany). Gel electrophoresis was carried on the NuPAGE® Novex® Bis-Tris gel system (Invitrogen AB, Lidingo, Sweden), and proteins were transferred to a polyvinylidene difluoride membrane using a Trans-Blot SD semi-dry transfer cell (Bio-Rad Laboratories AB, Uppsala, Sweden). After saturating 25 min with 5% milk in PBS (0.1% Tween 20), the membranes were incubated with primary (overnight, 4°C) and secondary (1 h, room temperature) antibodies. Membranes were washed three times 10 min in PBS (0.1% Tween 20) after incubations with each antibody. The bands were detected by enhanced chemiluminescence (ECL). Densitometry analysis of WB band intensities was performed using the image lab software version 3.0 on the molecular imager gel doc XR+ system (Bio-Rad Laboratories, Sweden). Primary antibodies: Polyclonal anti-mPGES-1, anti-mPGES-2 anti-cPGES, anti-COX-1 and anti-15-PGDH and monoclonal anti-COX-2 antibodies were purchased from Cayman chemicals, MI, USA. The anti-GAPDH antibodies were from Abcam. Secondary antibodies: the HRP-coupled anti-rabbit and anti-mouse antibodies were from GE healthcare, Sweden.

### Cytometric bead array for detection of cytokine production

Proinflammatory cytokine/chemokine production (IL-6, IL-8, RANTES, MCP-1, TNF-α, IL-12, IL-10, IFN-γ, IFN-α and IP-10) was determined using Inflammatory or Flex bead flow CBA (B&D Biosciences, Pharmingen, San Diego, CA, USA) and analysed according to the manufacturer's instructions.

### Statistics

Data are expressed as the mean ± SEM. One-way analyses of variance (ANOVA) followed by the Tukey–Kramer test for multiple comparisons were used to compare the treatment groups. P values less than 0.05 were considered significantly. For Figure 2, statistics were performed on mean area under the curve values for each treatment group. All statistics were performed using Prism (GraphPad Software, version 4).

## Results

### IL-1β and HMGB1 act synergistically to trigger PGE_2_ production in synovial fibroblasts from arthritic patients

To elucidate whether HMGB1 could contribute to the inflammatory process seen in arthritis via the induction of PGE_2_ synthesis, we investigated the effect of IL-1β_low_/HMGB1 complexes on synovial fibroblasts.

**Figure 1:**
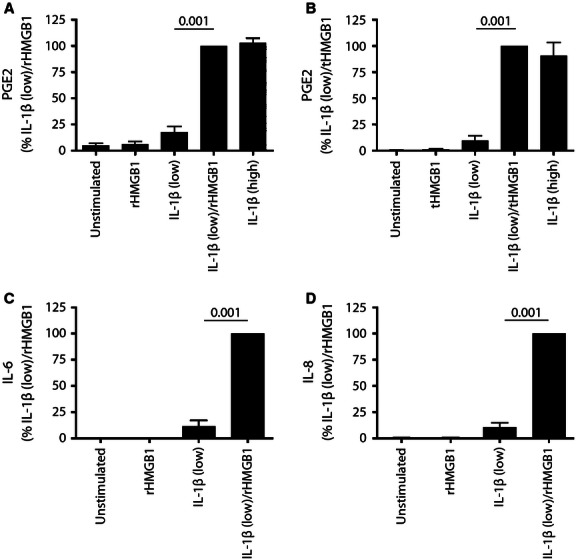
Low-concentration IL-1ß (IL-1ßlow) in combination with rat recombinant (r) or calf thymus-extracted (t)HMGB1 induces PGE2 and cytokine production. SFs were cultured in the presence of IL-1ß and HMGB1 separately or in complex. PGE2 production and cytokine release were measured in supernatants after 24 h of stimulation. (A) PGE2 production obtained when using rHMGB1 (*n* = 12) (B) PGE2 production obtained when using tHMGB1 (*n* = 4). (C) IL-6 release (rHMGB1) (*n* = 3) D) IL-8 release (rHMGB1) (*n* = 3). (A–D) results are expressed as per cent of the IL-1ßlow/r/tHMGB1 response to account for interpatient variability (mean ± SEM). PGE2 was measured by EIA and cytokines by CBA. P values were calculated by parametric ANOVA (Tukey–Kramer *post hoc* test).

SFs were cultured for 24 hours in the presence of thymus-extracted or recombinant HMGB1 (100 ng/ml) and low-concentration IL-1β (IL-1β_low_, 0.05 or 0.5 ng/ml) alone or in combination with HMGB1. Thymus-extracted or recombinant HMGB1 alone did not induce PGE_2_ production by SFs, while a slight increase could be detected in response to low concentrations of IL-1β (Fig. 1A,B). When IL-1β_low_ was combined with either recombinant or thymus-extracted HMGB1, PGE_2_ levels were significantly increased (*P* < 0.001) and reached the PGE_2_ levels induced by high-concentration (5 ng/ml) IL-1β (IL-1β_high_) (Fig. 1A,B). IL-1β_low_/HMGB1 complexes could also exert a synergistic effect on IL-6 and IL-8 production (Fig. 1C,D). The data are expressed as per cent of the response obtained with IL-1β_low_/HMGB1 complexes to account for interpatient variability. However, the PGE_2_ and cytokine responses in absolute units (for three representative patients) can be viewed in Figure S1.

### The kinetics of IL-1βlow/HMGB1-induced PGE2 and cytokine/chemokine production in SFs

It has earlier been established that IL-1β stimulation induces production of PGE_2_ and proinflammatory cytokines from synovial fibroblasts 32. Therefore, our next aim was to investigate whether the kinetics of PGE_2_ or cytokine production/profile induced in SFs were influenced by the action of IL-1β_**low**_/HMGB1 complexes.

**Figure 2:**
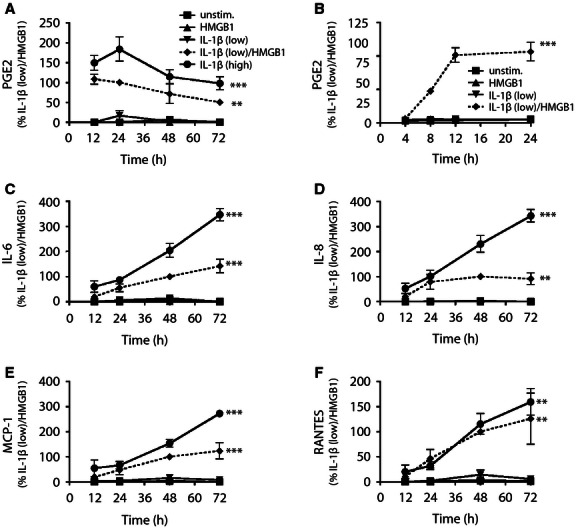
The PGE2 production induced by the IL-1βlow/HMGB1 complexes precedes cytokine release. SFs were cultured in the presence of IL-1β alone or in complex with HMGB1. PGE2 (A-B) and cytokine/chemokine (C-F) production was measured in supernatants after 4–72 h of stimulation. Results are expressed as per cent of IL-1βlow/HMGB1 (at 24 h for PGE2 and at 48 h for cytokines/chemokines) to account for interpatient variability. Data are expressed as means ± SEM from three separate experiments, and *P* values were calculated by parametric ANOVA. The Tukey–Kramer *post hoc* test was used to compare the HMGB1/IL-1βlow and IL-1βhigh treatment groups to the IL-1βlow treatment group.**P < 0.01, ***P < 0.001

Fibroblasts were incubated for 4-72 h with rHMGB1 and IL-1β_low_ alone or in combination, or with IL-1β_high_. HMGB1 alone or the suboptimal IL-1β_low_ concentration did not induce any PGE_2_ or cytokine/chemokine production when compared to unstimulated cells (Fig. 2A–E). In response to the IL-1β_low_/rHMGB1 treatment, PGE_2_ was shown to be elevated already after 12 h, with plateau levels between 12 and 24 h after which it slowly declined (Fig. 2A). A shorter incubation time revealed elevation was initiated between 4 and 8 h post-stimulation (Fig. 2B). In response to the IL-1β_high_ treatment, PGE_2_ levels were enhanced at 12-24 h and slowly decreased after 24 h. Next, we studied the kinetics of cytokine production from synovial fibroblasts. IL-1β_low_/rHMGB1-induced IL-8, IL-6, MCP-1 and RANTES were detectable after 12 h of stimulation and peaked at 48–72 h. A similar profile was observed with the IL-1β_high_-induced cytokine/chemokine production (Fig. 2C–F). Neither IL-1β_low_/rHMGB1 nor IL-1β_high_ alone induced TNF-α, IL-12, IL-10, IFN-γ, IFN-α or IP-10 (IL-1β) production by synovial fibroblasts.

Thus, the IL-1β_low_/HMGB1 complexes induced PGE_2_ and the cytokines/chemokines (IL-8, IL-6, MCP-1 and RANTES) with similar time kinetics as the IL-1β_high_ stimulation alone. Nevertheless, IL-1β_high_ gave a more sustained induction of IL-6, IL-8 and MCP-1 after 48–72 h of stimulation.

### IL-1βlow/HMGB1 induces the expression of the COX-2/mPGES-1 axis

**Figure 3:**
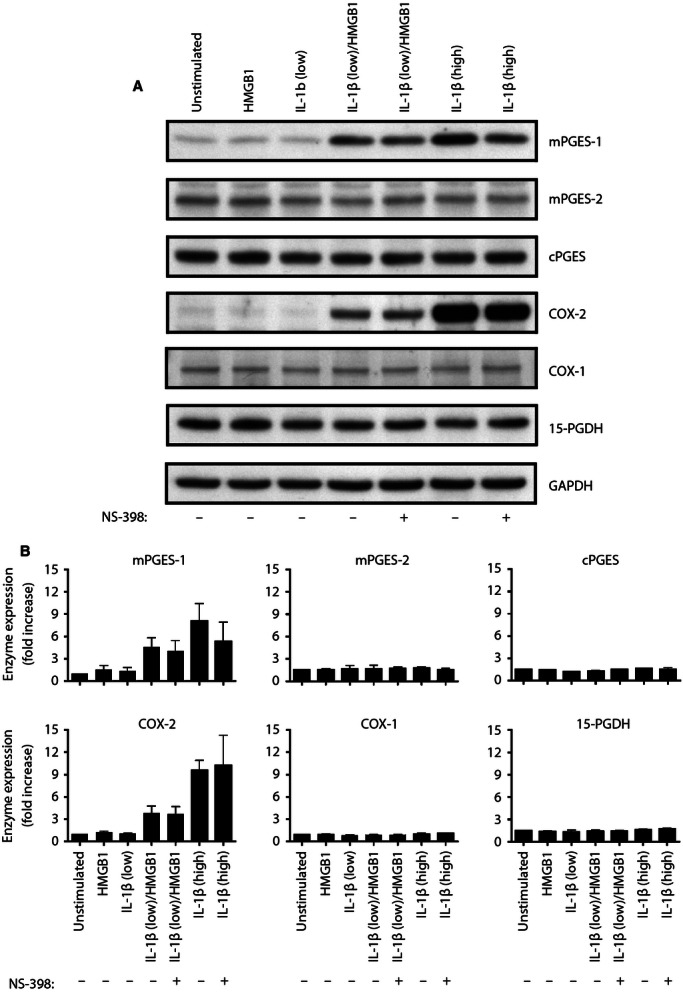
IL-1ßlow/HMGB1 induces the expression of the COX-2 and mPGES-1. SFs were cultured in the presence of IL-1ß alone or in complex with HMGB1. (A) The expression of mPGES-1/2, cPGES, COX-1/2, 15-PGDH and GAPDH was determined by Western Blot after 24 h of stimulation. (B) Densitometry plots showing protein expression normalized to GAPDH and expressed as fold increase relative to unstimulated cells levels. Densitometry data represent means ± SEM from three separate experiments.

Next, we investigated whether IL-1β_low_/HMGB1 complexes modulated the expression of enzymes of the PGE_2_ pathway in the same fashion as the IL-1β_high_ stimulation alone. SFs were cultured for 24 h in the presence of rHMGB1 and IL-1β_low_ alone or in complex or IL-1β_high_ alone. Cells were harvested and the expression of PGE_2_ pathway enzymes was investigated by Western blot. Unstimulated SFs showed low expression levels for the various enzymes analysed (Fig. 3A). When rHMGB1 and IL-1β_low_ were administered alone, all enzyme expression remained unchanged. When combined, however, the two stimuli triggered a noticeable upregulation in COX-2 and mPGES-1 expression, as can be visualized by a densitometry analysis (Fig. 3B). This explains the increase in PGE_2_ depicted in Fig. 1(A,B). No change could be recorded in the expression of mPGES-2, cPGES, COX-1 or 15-PGDH. The same pattern was observed in IL-1β_high_-stimulated SFs (Fig. 3A), thus suggesting that HMGB1 acts as an enhancer of IL-1β signalling pathway. No consistent effect on enzyme expression could be detected when COX-2 inhibitor NS-398 100 nm was added to IL-1β_low_/HMGB1 and IL-1β_high_–stimulated cells. PGE_2_ synthesis was completely inhibited by NS-398 (data not shown).

### Specific blockage of COX-2 enzymatic activity inhibits IL-1βlow/HMGB1-induced PGE2 synthesis and modulates cytokine production from SFs

**Figure 4:**
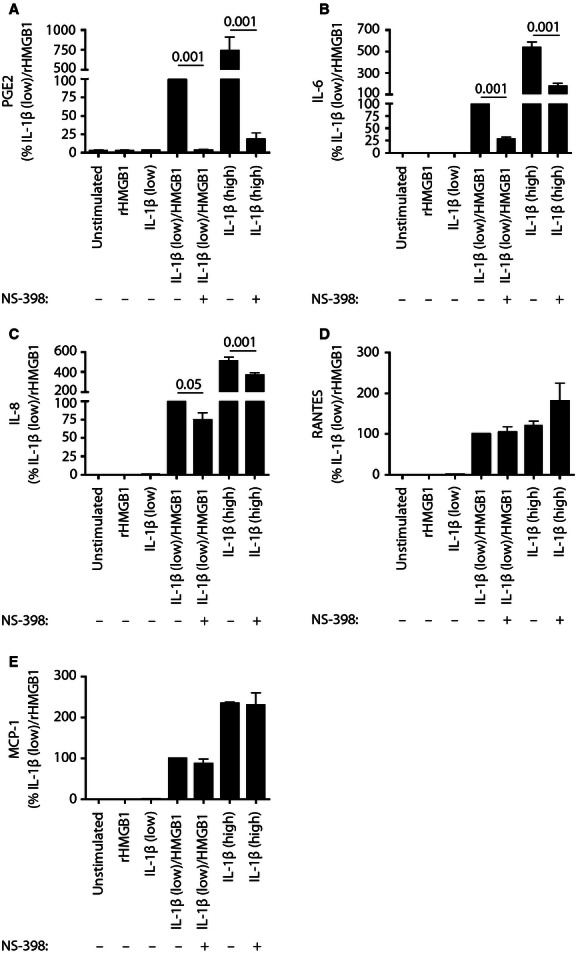
Specific blockage of COX-2 enzymatic activity inhibits IL-1*β*_low_/HMGB1- induced PGE_2_ synthesis and reduces cytokine production from RASFs. Specific blockage of COX-2 enzymatic activity inhibits IL-1*β*_low_/rHMGB1-induced PGE_2_ and reduces cytokine production from SFs. SFs were cultured in the presence of IL-1*β* alone or in complex with HMGB1 for 24 h. NS-398 was added at *t* = 0. Bar diagrams show the impact of COX-2 inhibition (NS-398 treatment) on (A) PGE2 and (B–E) cytokine production by SFs. Results are from three separate experiments and expressed as percent of the IL-1*β*_low_/rHMGB1 response to account for interpatient variability (mean ± SEM). PGE_2_ was measured by enzyme immunoassay and cytokines by CBA. *P* values were calculated by parametric anova (Tukey-Kramer *post hoc* test).

**Figure 5:**
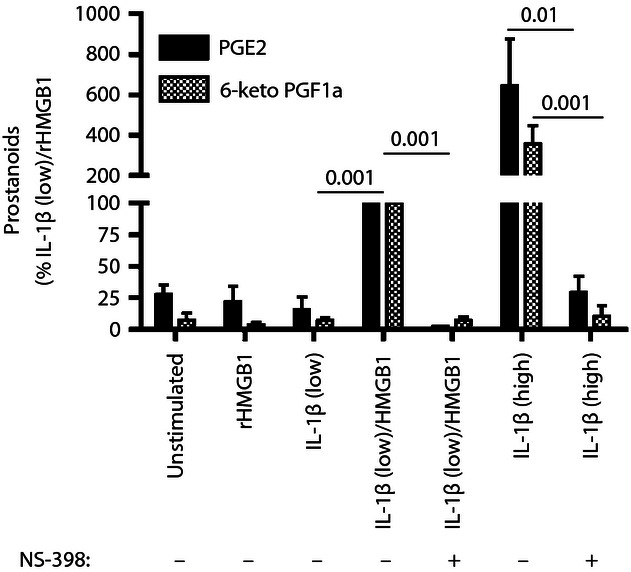
Prostanoid profile elicited by IL-1βlow/HMGB1 and IL-1βhigh and its modulation by COX-2 inhibition. SFs were cultured in the presence of IL-1β alone or in complex with HMGB1 for 24 h. NS-398 (0.1 μM) was added at *t* = 0. Bar diagrams show the impact of COX-2 inhibition (NS-398 treatment) on the prostanoid profile. Prostanoids were measured by LC-MS/MS. Results from three separate LC-MS/MS analyses (*n* = 3) are expressed as per cent of the IL-1βlow/rHMGB1 response to account for interpatient variability (mean ± SEM). *P* values were calculated by parametric ANOVA (Tukey–Kramer *post hoc* test).

We also investigated whether prostaglandin production was differentially modulated in the presence of HMGB1 by using the selective COX-2 inhibitor NS-398 and by studying the prostanoid profile elicited by IL-1β_low_/rHMGB1 complexes in SFs stimulated for 24 h. Using LC-MS/MS, the prostanoids PGE_2_, PGI_2_; PGD_2_, TxB_2_ and PGF_2a_ were analysed in cell supernatants. The PGE_2_ production triggered by IL-1β_low_/rHMGB1 and IL-1β_high_ was significantly inhibited by the presence of 0.1 μm NS-398 (Fig. 4A). When we analysed the prostanoid profile of SFs treated with rHMGB1 or IL-1β_low_, no modulation of prostanoid production could be detected. IL-1β_low_/rHMGB1 and IL-1β_high,_ however, gave rise to similar prostanoid profiles with PGE_2_ and PGI_2_ (6-keto PGF_1α_) clearly upregulated. The other primary prostanoids (PGD_2_, TxB_2_, PGF_2α_) remained under quantification limits. Treatment with NS-398 inhibited PGE_2_ and PGI_2_ synthesis induced by both stimuli (Fig. 5).

Next, we studied the effect of NS-398 on cytokine/chemokine production. A reduction in IL-6 and IL-8 production was noted, suggesting that the induction of cytokine/chemokine production by the complexes and IL-1_high_ are at least partly modulated by prostanoids (Fig. 4B,C). There was no significant change in RANTES or MCP-1 levels (Fig. 4D,E).

### IL-1β_low_/HMGB1 utilizes IL-1RI signalling for the induction of PGE_2_ production

**Figure 6:**
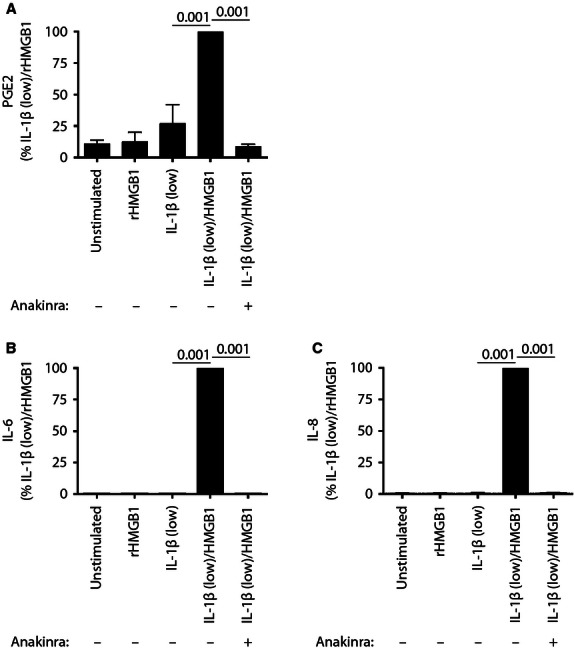
The IL-1ßlow/HMGB1 combina-tion signals through the IL-1RI receptor to upregulate PGE2 and cytokine production. SFs were cultured in the presence of IL-1ß alone or in complex with HMGB1. IL-1RI receptor antagonist, Anakinra, was added 1–2 h before stimulation of inflammation at 5 μg/ml. A) PGE2 and B,C) cytokine response were measured in supernatants after 24 h of stimulation. Results are expressed as per cent of the IL-1ßlow/HMGB1 response to account for interpatient variability. Data points represent means ± SEM from four separate experiments, and *P* values were calculated by parametric ANOVA test (Tukey–Kramer *post hoc* test).

Our next aim was to investigate whether the PGE_2_ production induced by IL-1β_low_/HMGB1 was mediated through IL-1RI. After 24 h of stimulation, a significant increase in PGE_2_ was detected in IL-1β_low_/rHMGB1-stimulated cells. Most importantly, when anakinra (5 μg/ml) was added 1–2 h prior to stimulation, it maintained PGE_2_ at unstimulated levels. (Fig. 6A) These results indicate that IL-1β_low_/HMGB1 complexes signal exclusively through IL-1RI to increase PGE_2_ synthesis (Fig. 6A) The same pattern was recorded for the production of cytokines IL-6 and IL-8, supporting our previous findings (Fig. 6B)^24^.

## Discussion

RA therapy has gone through great advances with the coming of biologics. These drugs are directed towards specific inflammatory processes targeting cytokines or immune cells. Still, none of those treatments, alone or in combination with DMARDs can stop, halt or reverse RA progression in all patients. It implies that additional signalling cascades, equally relevant to disease progression, can remain unaffected by the current antirheumatic treatments. One of the important pathways in the pathogenesis of RA is the PGE_2_ biosynthesis pathway, which is induced via multiple mechanisms and not properly targeted by methotrexate, TNF-α blockers or B cell depletion therapy 6–8. Interestingly, HMGB1 release, clearly induced in the synovium of RA patients, also remains unaltered by treatment with TNF-α blockers 26. This suggests a possible connection between HMGB1 release and induction of the PGE_2_ pathway in the RA synovium. Indeed, a link has already been drawn between HMGB1 and the PGE_2_ pathway in a study on the involvement of HMGB1 in the pathology of atherosclerosis. It showed that HMGB1 could trigger the PGE_2_ pathway in IL-1β-sensitized vascular smooth muscle cells 33. In this study, we investigated whether HMGB1 in complex with IL-1β could activate the PGE_2_ biosynthetic pathway in SFs.

Synovial fibroblasts isolated from patients are one of the reference cell systems used to study the molecular pathways involved in RA. In disease, RASFs mediate both cartilage and bone destruction and contribute to the chronic inflammatory loop that characterizes RA. They do so via the production of numerous cytokines (TNF-α, IL-1β, IL-8, IL-15, IL-22) and chemokines (MCP-1, MIP-1α, MIP-3α), as well as prostaglandins 34.

Recent advances in the field of HMGB1 research have revealed that redox modifications strongly influence the functional properties of the protein; that is, to conduct cell migration functions, an all-thiol form of HMGB1 is necessary, whereas cytokine induction is mediated by a partly oxidized HMGB1 isoform. Both these isoforms, in addition to the totally oxidized HMGB1 isoform, are likely to be present within the inflamed synovial tissue 35, 36. Thus, it is important to point out that the feature of HMGB1 studied in this article – its ability to enhance cytokine and prostanoid production by complex formation with IL-1 – is independent of its redox form.

Furthermore, we and others have demonstrated that, irrespective of its redox state, HMGB1 can mediate inflammation through complex formation with other endogenous (IL-1α and IL-1β) and exogenous (LPS, PAM_3_CSK_4_) molecules 12, 14, 15, 17, 29. Thus, HMGB1 can influence inflammatory processes both through a direct action and indirectly via complex formation. In this study, we have characterized the impact of complexes formed between HMGB1 and IL-1 on the prostanoid cascade with emphasis on the PGE_2_ pathway.

We used non-cytokine-inducing HMGB1 and suboptimal concentrations of IL-1β. The chosen concentrations for both molecules were physiologically relevant to the RA joint 37, 38. We demonstrated that HMGB1 did not affect PGE_2_ production by SF while the effect of low IL-1β concentrations was weak. However, HMGB1 in combination with the suboptimal concentration of IL-1β markedly induced PGE_2_ production by SFs (Fig. 1). The significant upregulation in PGE_2_ production was determined using both endogenous calf thymus-extracted HMGB1 (Fig. 1A) and recombinant HMGB1 produced in *E. coli* bacteria (Fig. 1B). These data suggest that HMGB1 might potentiate PGE_2_ production by SF in the presence even minute levels of IL-1β at the inflammatory site.

PGE_2_ levels are elevated in the synovia of RA patients, and synovial fibroblasts have been shown to upregulate the expression of mPGES-1 and COX-2 following stimulation with IL-1β (1). We studied the expression of various enzymes of the PGE_2_ pathway after stimulations with IL-1β_low_/HMGB1 complexes to determine how HMGB1 upregulates PGE_2_ synthesis. We found that IL-1β_low_/HMGB1 complexes trigger a rise in PGE_2_ through the upregulation of mPGES-1 and COX-2. They have no effect on COX-1, the other PGE synthases (mPGES-2, cPGES) or 15-PGDH, the PGE_2_ degrading enzyme. The complexes modulate the expression of the PGE_2_ synthesis cascade enzymes in the same manner as the stimulation with high-concentration IL-1β alone (Fig. 3). Moreover, we analysed the prostanoid profiles in the supernatants from the same cells. PGE_2_ and PGI_2_ were the major prostanoids upregulated by both IL-1β_low_/HMGB1 complexes and IL-1_high_ (Fig. 5). Together, the similar kinetics of PGE_2_ production, PG profiles and the induction of mPGES-1/COX-2 axis suggest that IL-1β_low_/HMGB1 complexes mediate their effects on the prostanoid cascade through the same pathway as IL-1β, via the IL-1RI receptor.

Further, we studied whether the induction of the PGE_2_ synthesis cascade and the cytokine response by IL-1β_low_/HMGB1 complexes were mediated via IL-1RI using the IL-1Ra. The addition of IL-1Ra to the SF cultures completely abolished the PGE_2_ response verifying that the induction of the PGE_2_ synthesis cascade by IL-1β_low_/HMGB1 complexes was dependent on IL-1RI signalling. Treatment with IL-1Ra also shut down the IL-6 and IL-8 responses, in line with our previous publication 24.

We also characterized the kinetics of production/release for PGE_2_ and several cytokines after stimulation with IL-1β_**low**_/HMGB1 complexes. We found that the PGE_2_ response was faster than the cytokine response, climaxing at 24 h. The release of IL-6, IL-8, MCP-1 and RANTES increased steadily up to 72 h, while no change in TNF-α, IL-12, IL-10, IFNγ, IFNα or IP-10 (IL-1β) could be detected. This is in line with the proinflammatory phenotype arising from the stimulation of SFs with high-concentration IL-1β alone (Fig. 2).

NS-398 was employed to study the repercussions of COX-2 inhibition on the prostanoid profile and cytokine release in SFs stimulated with IL-1β_low_/HMGB1 complexes. The PGE_2_ response triggered by complexes was completely inhibited by NS-398 (Figs. 4 and 5). The rise in PGE_2_ and 6-keto PGF_1α_ triggered by complexes as seen on prostanoid profile was also inhibited by NS-398, confirming that HMGB1's effects on the PGE_2_ pathway are mediated through the induction of COX-2. Lastly, we investigated the effect of the NS-398 treatment on cytokine production. IL-6 and IL-8 were found to be significantly inhibited, depicting an anti-inflammatory effect of COX-2 inhibition on HMGB1-stimulated SFs. The impact of COX-2 inhibition on cytokine production indicates that IL-1β_low_/HMGB1 complexes did induce/modulate cytokine production in part through prostanoid synthesis.

In conclusion, we have demonstrated that HMGB1 can promote the induction of mPGES-1 and COX-2 and production of PGE_2_, through potentiation of the IL-1β response in SFs. The amplification of the PGE_2_ biosynthesis pathway by HMGB1 might constitute an important pathogenic mechanism perpetuating inflammatory and destructive activities in RA.

Therefore, targeting HMBG1 or mPGES-1 could complement the current therapies in the treatment of rheumatoid arthritis.

## Supporting Information

Figure S1. PGE2 and cytokine response to IL-1blow/HMGB1 complexes in three representative patients to depict inter-patient variability. SFs were cultured in the presence of IL-1b and HMGB1 separately or in complex. PGE2 production and cytokine release were measured in supernatants after 24 h of stimulation. Mediator production obtained when using rHMGB1 (A) PGE2 B) IL-6 C) IL-8 D) MCP-1 E) RANTES. PGE2 was measures by EIA and cytokines by CBA. Data is expressed in pg/ml.

## References

[1] Stichtenoth DO, Thoren S, Bian H, Peters-Golden M, Jakobsson PJ, Crofford LJ (2001). Microsomal prostaglandin E synthase is regulated by proinflammatory cytokines and glucocorticoids in primary rheumatoid synovial cells. J Immunol.

[2] Thoren S, Jakobsson PJ (2000). Coordinate up- and down-regulation of glutathione-dependent prostaglandin E synthase and cyclooxygenase-2 in A549 cells. Inhibition by NS-398 and leukotriene C4. Eur J Biochem.

[3] Simon LS, Weaver AL, Graham DY (1999). Anti-inflammatory and upper gastrointestinal effects of celecoxib in rheumatoid arthritis: a randomized controlled trial. JAMA.

[4] Jakobsson PJ, Thoren S, Morgenstern R, Samuelsson B (1999). Identification of human prostaglandin E synthase: a microsomal, glutathione-dependent, inducible enzyme, constituting a potential novel drug target. Proc Natl Acad Sci USA.

[5] Westman M, Korotkova M, af Klint E (2004). Expression of microsomal prostaglandin E synthase 1 in rheumatoid arthritis synovium. Arthritis Rheum.

[6] Korotkova M, Westman M, Gheorghe KR (2005). Effects of antirheumatic treatments on the prostaglandin E2 biosynthetic pathway. Arthritis Rheum.

[7] Gheorghe KR, Thurlings RM, Westman M (2011). Prostaglandin E2 synthesizing enzymes in rheumatoid arthritis B cells and the effects of B cell depleting therapy on enzyme expression. PLoS ONE.

[8] Gheorghe KR, Sadique S, Leclerc P (2012). Limited effect of anti-rheumatic treatment on 15-prostaglandin dehydrogenase in rheumatoid arthritis synovial tissue. Arthritis Res Ther.

[9] Castiglioni A, Canti V, Rovere-Querini P, Manfredi AA (2011). High-mobility group box 1 (HMGB1) as a master regulator of innate immunity. Cell Tissue Res.

[10] Yang H, Hreggvidsdottir HS, Palmblad K (2010). A critical cysteine is required for HMGB1 binding to Toll-like receptor 4 and activation of macrophage cytokine release. Proc Natl Acad Sci USA.

[11] Kazama H, Ricci JE, Herndon JM, Hoppe G, Green DR, Ferguson TA (2008). Induction of immunological tolerance by apoptotic cells requires caspase-dependent oxidation of high-mobility group box-1 protein. Immunity.

[12] Ivanov S, Dragoi AM, Wang X (2007). A novel role for HMGB1 in TLR9-mediated inflammatory responses to CpG-DNA. Blood.

[13] Urbonaviciute V, Furnrohr BG, Meister S (2008). Induction of inflammatory and immune responses by HMGB1-nucleosome complexes: implications for the pathogenesis of SLE. J Exp Med.

[14] Youn JH, Kwak MS, Wu J (2011). Identification of lipopolysaccharide-binding peptide regions within HMGB1 and their effects on subclinical endotoxemia in a mouse model. Eur J Immunol.

[15] Tian J, Avalos AM, Mao SY (2007). Toll-like receptor 9-dependent activation by DNA-containing immune complexes is mediated by HMGB1 and RAGE. Nat Immunol.

[16] Schiraldi M, Raucci A, Munoz LM (2012). HMGB1 promotes recruitment of inflammatory cells to damaged tissues by forming a complex with CXCL12 and signaling via CXCR4. J Exp Med.

[17] Hreggvidsdottir HS, Ostberg T, Wahamaa H (2009). The alarmin HMGB1 acts in synergy with endogenous and exogenous danger signals to promote inflammation. J Leukoc Biol.

[18] Hamada T, Torikai M, Kuwazuru A (2008). Extracellular high mobility group box chromosomal protein 1 is a coupling factor for hypoxia and inflammation in arthritis. Arthritis Rheum.

[19] Kokkola R, Li J, Sundberg E (2003). Successful treatment of collagen-induced arthritis in mice and rats by targeting extracellular high mobility group box chromosomal protein 1 activity. Arthritis Rheum.

[20] Pisetsky DS, Erlandsson-Harris H, Andersson U (2008). High-mobility group box protein 1 (HMGB1): an alarmin mediating the pathogenesis of rheumatic disease. Arthritis Res Ther.

[21] Schierbeck H, Lundback P, Palmblad K (2011). Monoclonal anti-HMGB1 (high mobility group box chromosomal protein 1) antibody protection in two experimental arthritis models. Mol Med.

[22] Ostberg T, Kawane K, Nagata S (2010). Protective targeting of high mobility group box chromosomal protein 1 in a spontaneous arthritis model. Arthritis Rheum.

[23] Kokkola R, Sundberg E, Ulfgren AK (2002). High mobility group box chromosomal protein 1: a novel proinflammatory mediator in synovitis. Arthritis Rheum.

[24] Wahamaa H, Schierbeck H, Hreggvidsdottir HS (2011). High mobility group box protein 1 in complex with lipopolysaccharide or IL-1 promotes an increased inflammatory phenotype in synovial fibroblasts. Arthritis Res Ther.

[25] af Klint E, Grundtman C, Engstrom M (2005). Intraarticular glucocorticoid treatment reduces inflammation in synovial cell infiltrations more efficiently than in synovial blood vessels. Arthritis Rheum.

[26] Sundberg E, Grundtman C, Af Klint E (2008). Systemic TNF blockade does not modulate synovial expression of the pro-inflammatory mediator HMGB1 in rheumatoid arthritis patients–a prospective clinical study. Arthritis Res Ther.

[27] Bresnihan B, Alvaro-Gracia JM, Cobby M (1998). Treatment of rheumatoid arthritis with recombinant human interleukin-1 receptor antagonist. Arthritis Rheum.

[28] Paonessa G, Frank R, Cortese R (1987). Nucleotide sequence of rat liver HMG1 cDNA. Nucleic Acids Res.

[29] Wang H, Bloom O, Zhang M (1999). HMG-1 as a late mediator of endotoxin lethality in mice. Science.

[30] Sha Y, Zmijewski J, Xu Z, Abraham E (2008). HMGB1 develops enhanced proinflammatory activity by binding to cytokines. J Immunol.

[31] Glennas A, Thorsrud AK, Rugstad HE, Jellum E (1985). Mapping of proteins from cultured fibroblasts of synovial and subcutaneous origin by high resolution two-dimensional polyacrylamide gel electrophoresis. Ann Rheum Dis.

[32] Angel J, Berenbaum F, Le Denmat C, Nevalainen T, Masliah J, Fournier C (1994). Interleukin-1-induced prostaglandin E2 biosynthesis in human synovial cells involves the activation of cytosolic phospholipase A2 and cyclooxygenase-2. Eur J Biochem.

[33] Jaulmes A, Thierry S, Janvier B, Raymondjean M, Marechal V (2006). Activation of sPLA2-IIA and PGE2 production by high mobility group protein B1 in vascular smooth muscle cells sensitized by IL-1 beta. Faseb J.

[34] Huber LC, Distler O, Tarner I, Gay RE, Gay S, Pap T (2006). Synovial fibroblasts: key players in rheumatoid arthritis. Rheumatology (Oxford).

[35] Venereau E, Casalgrandi M, Schiraldi M (2012). Mutually exclusive redox forms of HMGB1 promote cell recruitment or proinflammatory cytokine release. J Exp Med.

[36] Yang H, Lundback P, Ottosson L (2012). Redox modification of cysteine residues regulates the cytokine activity of high mobility group box-1 (HMGB1). Mol Med.

[37] Taniguchi N, Kawahara K, Yone K (2003). High mobility group box chromosomal protein 1 plays a role in the pathogenesis of rheumatoid arthritis as a novel cytokine. Arthritis Rheum.

[38] Kahle P, Saal JG, Schaudt K, Zacher J, Fritz P, Pawelec G (1992). Determination of cytokines in synovial fluids: correlation with diagnosis and histomorphological characteristics of synovial tissue. Ann Rheum Dis.

